# Microdissection is the best way to perform sperm retrieval in men with non-obstructive azoospermy? | *Opinion: No, there are other options*

**DOI:** 10.1590/S1677-5538.IBJU.2018.06.03

**Published:** 2018

**Authors:** Marcelo Vieira

**Affiliations:** 1 Membro Titular da Sociedade Brasileira de Urologia, Rio de Janeiro, RJ, Brasil; 2 Urologista do Projeto ALFA, São Paulo, SP, Brasil

**Keywords:** Azoospermia, Microdissection, Sperm Retrieval, Fertility

In the last 23 years, Intracitoplasmic Sperm Injection (ICSI) has given non-obstructive azoospermic man the opportunity to become biological fathers, if sperm could be found in their testicles. These men present the biggest challenge in the routine of infertility clinics around the World, since there are no positive, clinical or laboratory, prognostic factors for sperm recovery. Once testicular sperm has been regularly used for ICSI, discussion about which technique for testicular sperm retrieval has been done. Sperm can be harvest from testicular parenchyma by: open biopsy (Testicular Sperm Extraction-TESE), percutaneous aspiration (Testicular Sperm Aspiration), open guided biopsy by previous cytology (Testicular fine-needle Aspiration) and open biopsy using microsurgery technique (Testicular Microdissection). The proposed techniques have the same objective, to find sperm with minimal testicular damage and in a reproducible way (1).

TESE can be done by a large longitudinal incision on the testicular albuginea and excision of a representative large testicular fragment where multiple tubule samples can be examined for sperm presence. A variation is a multiple biopsy approach by incision of multiple sites and searching for sperm in each fragment. Both techniques have similar results once spermatogenesis is diffusely distributed, but sometimes very sparse, making it difficult to find sperm by random biopsies (2).

Testicular fine needle aspiration (TFNA) had been defined for histologic testis examination, and brought to infertility use as a tool to identify spermatogenesis foci and guide sperm retrieval for ICSI. A simple procedure done under local anesthesia that correlates almost 90% with histology, although it is necessary a second intervention for sperm retrieval (3).

Testicular Microdissection described by Schlegel brought a new concept for sperm retrieval by using optical magnification to identify spermatogenesis foci based on the morphological testicular tubules characteristics and initial better results with minimum parenchyma amount excised (4).

The key questions are: what sperm recovery rate (SRR) is considered good and how to find spermatogenesis foci with minimal testicular damage? Multiple sampling, fine needle citology or magnification? The present techniques have positive and negatives aspects, but the debate about those aspects was interrupted after testicular microdissection, despite different experiences.

Sperm recovery rate for TESE varies among published data:Silber et al. 1997, 51%; Ostad et al. 1998, 58%; Tournaye 1999, 48%; Amer et al. 1999, 49%; Silber 2000, 55%; Bettella et al. 2005, 59%. All papers report SRR around 50%, and varieing according histologic findings (2, 5-9).

Testicular microdissection SRR was reported in a review from 42 to 63% and Schlegel's group showed 52% after 1,414 cases (10, 11).

Comparison between the different techniques is quite difficult because of the differences in histologic paterns, but published data shows an advantage for Microdisscetion TESE, although SRR for TESE in analized papers was bellow 50% (16.5-45%) (12, 13). A prospective study done by Ghalayini et al. showed significant difference in SRR for testicular microdissection compared with conventional TESE, but in other studies the median SRR for TESE was under 40%; this could be explained by taken only 3 samples from upper, median and lower testicular portion. FSH and testicular volume were prognostic factors for sperm retrieval adding some more discussion on the theme; papers differ on that opinion, and the classification chosen by the authors for FSH levels and testicular volume may have justified the results (14). One fundamental aspect discussed by authors was the fact that histological evaluation showed 61% of sperm on tissue retrieved for anatomical exam, enlightening the importance of micromanipulation laboratory for better SRR.

Microdissection TESE also have showed published irregular results as reported in one study conducted in Japan between 2014-2015 with 83% response rate from 47 infertility centers in the country analyzing the treatment results of 7,268 patients. Azoospermia was present in 1185 patients. Conventional TESE was performed in 231 patients with 98.3% sperm retrieval rate (SRR) and 56.2% pregnancy rate. Testicular microdissection was performed in 695 patients with 34% SRR and 11.8% pregnancy rate. The question with these data is the absence of clear azoospermia classification, once they showed good results for conventional TESE, probably because they were treating obstructive azoospermia. The most important conclusion about these data was the low SRR for testicular microdissection among Japanese certified specialists revealing some difficulty in finding classic dilated seminal tubules which sustains spermatogenesis (15).

Testicular damage is caused by injury of sub albugineal vessels and may be verified by symptoms, ultrassonographic changes and hormonal levels. Ultrasonographic evaluation after TESE showed parenchyma hematomas and acute inflammatory alterations (82% and 64% after 3 and 6 months respectively) and 2 patients complained about unilateral testicular atrophy (3%); unfortunately about 50% of the initial patients had not the ultrasonography done (16). Post TESE testosterone levels data were unconclusive in two studies showing divergents results (17, 18). The excision of a large sample or multiple biopsies are hypothetic more harmful to sub albugineal arteries and the use of magnification may avoid the damage and subsequently scars and testicular atrophy, but requires surgical microscope (2). TFNA also can diagnose sperm in testicular parenchyma, with minimal damage showed by immediate post-operative ultrasound and with a good cytology/histology correlation but demands a second intervention for sperm recovery (3).

We propose a different technique that was inspired on testicular fine needle aspiration (TFNA) together with TESE. The idea is to associate mapping from TFNA and the better amount of tissue for analysis provided by TESE, with no need for a second **procedure on ICSI day, that we called Open Testicular Mapping (OTEM)**. Under sedation and cord block the testicle is delivered through a median scrotal incision and multiple testicular punctures are made in the tunica albuginea using a 19-gauge needle. The needle is used to open a tiny hole in the tunica. With compression of the testicle a portion of testicular tissue protrudes and is pulled out with the help of two microsurgical tweezers. The testicular samples are placed on a sterile Petri dish containing 0.6 mL of culture medium, minced with micro scissor and finally passed through a 24-gauge angiocatheter. The analysis is done by FIV laboratory personal under inverted microscope using 400 X magnification for sperm presence after each collection; if enough sperm to inject oocytes is found, the procedure is concluded. If no sperm is found, a new hole is performed with a new testicular tissue sampling. Six holes are made on upper, middle and lower portion of the testis, when necessary. One sample from the middle portion is sent for histological examination. OTEM iss usually performed the afternoon before ovary aspiration, first on the right testis, upper portion, from medial to lateral, except when left testis is larger or the right absent. If no sperm are found on the first testis, the contra lateral is approached in the same way.

We presented at European Society of Human Reproduction and Embryology 2018 meeting a retrospective study of patients presenting with non-obstructive azoospermia from 2008 to 2016 evaluating 92 patients submitted to OTEM and ICSI. SRR was 54% (50/92). The most frequently found histologic pattern was maturation arrest (43), followed by Sertoli cell only (23), hypospermatogenesis (15) and testicular atrophy (11). SRR for each pattern was 48% (21/43) for maturation arrest, 43% (10/23) for Sertoli cell only, 86% (13/15) for hypospermatogenesis and 54% (6/11) for testicular atrophy.

We are comfortable in offer to our patients a SRR above 50%, theoretically with minimal vascular damage once the albuginea isn't open and without extended parenchyma dissection. The tactic of use of a needle for perforation in a non-vascularized area and not a tunica incision may prevents hematomas and parenchyma's devascularization. We are confident that for better sperm retrieval the extrusion of a group of tubules and pulling them out entirely is preferred rather than a multiple cross tubule section. We do hope to enhance our experience for large sample results for publishing.
